# From authoring to editing: a competency framework for trainee use of ambient AI clinical documentation

**DOI:** 10.1038/s41746-026-03351-5

**Published:** 2026-10-12

**Authors:** Yash Raka, Reed Van Deusen, Holly Wiberg

**Affiliations:** 1 University of Pittsburgh School of Medicine, Pittsburgh, PA USA; 2 Heinz College of Information Systems and Public Policy, Carnegie Mellon University, Pittsburgh, PA USA

**Keywords:** Business and industry, Computational biology and bioinformatics, Health care, Mathematics and computing

## Abstract

Ambient AI has spread through clinical settings faster than curricula can adapt, shifting trainee documentation from authoring to editing notes. Authoring historically honed clinical reasoning for trainees, yet medical education is not prepared to develop the editing task now replacing it. We define this task as a teachable competency with four domains - error detection, clinical reconciliation, independent generation, and trust calibration - and describe how simulation can teach them.

## Introduction

Ambient artificial intelligence (AI) scribes have achieved rapid and widespread clinical adoption, with early evidence suggesting trainees derive disproportionate benefit compared to other clinician groups. Yet the outcomes measured so far - documentation time, cognitive workload, clinician burnout - speak to the value of these tools for workforce sustainability rather than for medical education. Physician practice is shifting from authoring clinical notes to editing AI-generated ones, and trainees are inheriting that shift without a defined competency for performing it. Here, we argue that reviewing AI-generated clinical documentation is a teachable competency that current curricula are not yet designed to develop, and that simulation-based education is well-suited to both teach the competency and produce the developmental evidence the clinical literature has yet to produce.

## The trainee benefit

Ambient AI scribes - tools that passively capture clinician-patient conversations and generate draft clinical notes - are among the most rapidly-adopted generative AI applications in healthcare, documenting more than 2.5 million clinical encounters in a single year at one integrated system^1^.

The most substantial real-world evaluation to date of this tool’s impact tracked ambient AI scribe use across five academic health centers over more than 2 years^2^. Among 8581 clinicians, adopters saved a mean of 13 min in the electronic health record (EHR) per 8 h of scheduled time. Notably, resident physicians saved 94 min per 8 h - nearly seven times the pooled adopter mean. A Yale pilot with 47 trainees corroborated this trainee benefit, with NASA Task Load Index (NASA-TLX) cognitive workload scores improving from 56.3 to 43.3 after 2 months of ambient AI scribe use (*p* < 0.001), with 91.5% adoption^3^.

These findings are likely replicable. Trainees carry the highest documentation burden and the steepest cognitive load of any clinician group; it is unsurprising that they benefit substantially when that burden is reduced. The incentive structure now points in one direction: residency programs will adopt ambient AI scribes because the evidence says their residents will likely benefit. Medical students entering clerkships will encounter these tools as a standard workflow component.

Given the rapid pace of adoption, the question is not whether trainees will use ambient AI scribes. Rather, the question is whether medical education is preparing them to do so in a way that supports, rather than bypasses, their development as clinicians.

## What current evidence does not measure

Responsible deployment of ambient AI rests on a human-in-the-loop assumption: the physician reviewing AI-generated documentation will catch errors before they reach the medical record. For this model to hold, two things must be true: the reviewer must be competent at the review task, and the review must actually happen. The published evidence speaks to neither.

The deployment studies above, and ambient AI evaluation more broadly, focus on documentation time and provider burden^4–7^. These outcomes matter for workforce sustainability and physician well-being, but they do not measure whether clinicians are catching errors in AI-generated notes or whether the clinical reasoning that has historically developed through independent documentation is being preserved. The Yale pilot authors acknowledged this gap directly, noting the need for research on ambient AI’s impact on trainee learning and development^3^.

These concerns have been documented. Ohde and colleagues argued that the cognitive demands of reviewing LLM output have been underestimated, since humans follow the path of least effort and over-rely on automation aids^8^. Coiera and Fraile-Navarro have made the broader case that the field has been measuring the wrong outcomes - efficiency and burden rather than accuracy, safety, and learning^9^.

Meanwhile, ambient AI error rate data largely comes from validation studies, not real-world deployment. A systematic review found error rates exceeding 50% in conversational and multispeaker scenarios^10^. Comparative evaluations have found AI-generated notes score higher on thoroughness and organization but lower on accuracy and internal consistency relative to physician-authored notes^11^, with the most common error being omission rather than inaccurate insertions^12^.

The stakes are heightened with trainees. Both note quality and clinical reasoning are established during medical education, and trainees are most likely to break the human-in-the-loop assumption: they are still building the competence the review task demands, at the stage when offloading it is most tempting. Yet no study has measured whether the notes trainees sign off on are accurate, whether trainees are catching embedded errors, or whether the reasoning that independent documentation once built is being preserved, redirected, or displaced.

This gap is unlikely to close on its own. Clinical deployments lack the structured comparison conditions, controlled error exposure, and standardized measurement required to isolate developmental outcomes. The research the field has called for will require a different venue, which we turn to in proposing our framework below.

## From authoring to editing

Clinical documentation has historically served two functions in medical training. The first is the generation of the medical record itself. The second is the development of clinical reasoning: the cognitive work of prioritizing information, justifying decisions, and synthesizing fragmented data into a coherent clinical narrative.

As ambient AI increasingly performs the first function, the second is put at risk, because authoring and reviewing are not cognitively equivalent. Independent authoring engages the effortful “System 2” processing of synthesis and prioritization, whereas reviewing a fluent AI-generated note invites the fast, low-effort acceptance characteristic of “System 1”^13^. This distinction matters pedagogically. The generation effect establishes that information is better learned when actively produced than when merely reviewed^14^. Review also invites automation bias - the documented tendency of humans to over-rely on and under-scrutinize automated output^15^, a tendency Ohde and colleagues find is amplified by the cognitive demands of reviewing LLM-generated content^8^. This literature suggests that the reviewing competency must therefore be deliberately taught, because the editorial task does not naturally recruit the reasoning that authoring developed as a byproduct.

Yet medical education teaches trainees to write clinical notes from scratch, not to critically evaluate AI-generated documentation. Based on publicly available institutional materials, 13 of 25 U.S. medical schools receiving the highest NIH funding^16^ showed evidence of interest in AI education; 2 had formally integrated AI into the core MD curriculum, 2 had active pilots, and the remainder had no publicly documented curricular implementation at the time of review (May 2026). None of the identified curricula explicitly addressed ambient AI scribes.

Efforts to address ambient AI in training remain few and unstructured. An internal medicine residency pilot offers one of the first accounts from both resident and faculty perspectives, with 48 residents generating nearly 1000 clinical notes over 6 months^17^. Its authors proposed seven guardrails mapped to the Accreditation Council for Graduate Medical Education core competencies, observing that without intentional action, “trainees may become passive editors rather than active authors of care.” The field now needs to move from ad hoc exposure to defined competencies that can be taught, assessed, and scaled.

The editorial skills required for competent AI documentation review are specific and teachable, not innate extensions of clinical practice, and nothing in the current curriculum targets them directly. We define them below as four competency domains: detecting errors and omissions, judging whether the note reflects the clinical reasoning of the encounter, generating notes independently, and calibrating trust across contexts.

The educational pattern here is familiar. When EHRs were adopted across US medical systems, medical schools largely restricted student access and did not formalize training^18^. A systematic review published years later identified critical gaps in EHR education for both undergraduate and postgraduate learners^19^. The American Medical Association eventually called for structured EHR training^20^, after observing that inadequate preparation was affecting first-year residents. The rationale for curricular delay at the time - concerns about billing, note quality, and patient safety - mirrors current hesitation around ambient AI. The parallel is imperfect, since EHRs replaced a documentation system, while ambient AI augments one. Yet the educational pattern is identical: rapid clinical deployment, institutional caution in training, deferred curricular integration, and trainees entering practice unprepared. On a faster timeline and with higher developmental stakes, the field is at risk of repeating a known mistake.

## A competency framework for documentation review

Abdulnour and colleagues recently proposed DEFT-AI, a framework for structured clinical supervision of AI use in medical education that identifies three core risks to trainee development: *deskilling*, in which overreliance on AI erodes existing clinical reasoning abilities; *never-skilling*, in which trainees fail to develop essential competencies in the first place; and *mis-skilling*, in which trainees unconsciously adopt AI-generated errors and biases as their own^21^. DEFT-AI provides a helpful framework for curricular development, as demonstrated in a recent proposal surrounding AI-enabled diagnostic reasoning tools in medical education^22^.

If AI documentation review is a clinical skill, it can similarly be defined, taught, and assessed like any other. Thus, we propose a preliminary framework organized around four competency domains, each mapped to a DEFT-AI risk category (Table 1).

**Table 1:** Proposed competency framework for ambient AI documentation review in medical education, grounded in the DEFT-AI risk categories (Abdulnour et al.^21^)

Competency domain	DEFT-AI risk addressed	Training stage	Learning objective	Example educational exercise	Assessment metrics
Error detection	Never-skilling	Pre-clerkship (introduction); Clerkship (application)	Identify and correct factual errors, omissions, hallucinations, and misattributions in AI-generated clinical notes	After a simulated encounter with ambient AI active, review and correct AI-generated notes with planted errors (omissions, inaccuracies, hallucinations) at graduated difficulty	Error detection rate, correction accuracy tracked by error type
Clinical reconciliation	Never-skilling	Clerkship (introduction); Residency (refinement)	Evaluate whether the note reflects the clinical reasoning and prioritization of the encounter, discriminating relevant from irrelevant information	After a simulated encounter with ambient AI active, review AI-generated notes that are factually correct but misweighted in clinical priority or misordered in the presented reasoning; correct the notes and provide a written rationale for the edits	Learner justification for organizational edits to ambient AI note assessed via rubric; agreement score of reconciled note with reference standard
Independent generation	Deskilling	Clerkship through Residency (maintenance of established authoring)	Produce a clinically sound note without AI assistance; preserve the pedagogical function of documentation as a reasoning tool	Periodic “AI-off” exercises: write full H&P or progress note independently after standardized encounter	Faculty review using standard documentation rubric; longitudinal tracking of note quality
Trust calibration	Mis-skilling	Post-clerkship / Early residency	Appropriately calibrate reliance on AI output based on clinical context and known AI performance characteristics	Simulated encounters with mixed scenarios: scenarios where AI performs reliably (straightforward, single-speaker) and scenarios where AI underperforms (multispeaker, non-English, high-acuity, rare presentations); trainee decides whether to use, edit, or override; after each exercise, trainees rate their confidence in their review accuracy, then compare it against their measured error-detection performance	Appropriateness of trust decisions across scenario types; score override/acceptance patterns against expert consensus; confidence-accuracy calibration (agreement between self-rated confidence and measured performance)

Framework requires empirical validation DEFT-AI supervisory structure (Diagnosis, Evidence, Feedback, Teaching) applies to post-exercise debriefing across all domains.

*Error detection* (never-skilling). Can the trainee identify factual errors, omissions, hallucinations, and misattributions in AI-generated clinical notes? This domain targets the core editorial function: catching what the AI got wrong *and, as an editor rather than a passive reviewer, correcting it.* Training can begin with annotated sample notes containing planted errors and progress to real-time detection during simulated encounters.

*Clinical reconciliation* (never-skilling). Can the trainee determine whether an AI-generated note reflects the clinical reasoning of the encounter, not merely its facts? Where error detection verifies factual accuracy, reconciliation requires clinical judgment: evaluating whether the note prioritizes what matters - discriminating salient information from irrelevant detail, preserving the reasoning and rationale of the encounter, and reflecting how the assessment was actually constructed. A note can be factually correct yet still misrepresent the encounter by misweighting clinical facts in the narrative or sequencing findings in a way that implies a different diagnostic logic than what was performed. This reconciliation is distinct from the factual verification performed in error detection. This is a higher-order competency that requires clinical maturity beyond the pre-clerkship years, since a trainee cannot judge whether information is appropriately prioritized without knowing what is clinically relevant.

*Independent generation* (deskilling). Can the trainee produce a clinically sound note from scratch when AI is unavailable, inappropriate, or the patient does not consent? This domain preserves the pedagogical function of documentation as a reasoning tool. Periodic “AI-off” exercises ensure trainee reasoning does not become dependent on AI scaffolding. This domain concerns maintaining an authoring ability once developed, not forming it initially; the foundational authoring that first builds clinical reasoning precedes this framework and is presupposed by it.

*Trust calibration* (mis-skilling). Does the trainee appropriately calibrate reliance on AI output across clinical contexts? This addresses both over-trust - the automation complacency documented by Ohde and colleagues^8^ - and under-trust. Trust calibration is a behavioral skill: trainees must not only understand where AI scribes underperform (complex multispeaker encounters, non-English-language visits, high-acuity scenarios, unusual presentations) but also act on that understanding when deciding whether to use, edit, or override AI output. Calibration also has an internal dimension: trainees must monitor the limits of their own review, not only the AI’s. Comparing their confidence in a review against their measured accuracy—rating how thoroughly they believe they caught errors, then checking that against how many they actually caught—builds awareness of personal vulnerability to missed AI errors and makes trust calibration a self-regulatory skill rather than static knowledge of where AI fails. By training trainees to systematically interrogate AI output against their own clinical reasoning, this domain also protects against passive internalization of AI-generated errors and biases by making calibration explicit.

The learning objectives, simulation exercises, and assessment metrics for each domain are specified in Table 1. Implementation should follow a cognitive progression recommended by cognitive load theory^23^. The proposed sequencing across domains reflects this progression: error detection is a lower-load, fact-anchored task and thus is introduced before clinical reconciliation, which requires clinical judgment to evaluate weighting and priority on top of accuracy. Trust calibration is the culminating step, which presupposes a reliable baseline for what accurate review looks like. Within each domain, difficulty should be gradually increased: error detection, for instance, can begin with planted errors of a single discrete type against a known reference, advancing to more subtle or complex error types once basic skills are solidified. Finally, all competencies should be treated as sustained skills rather than one-time introductions through spaced repetition^24^; for example, distributing “AI-off” authoring at regular intervals across training rather than concentrating it in a single block.

These design principles require a venue that can stage them. Simulation-based education is structurally suited to serve a dual function. As a teaching venue, simulation centers provide controlled environments in which AI scribes can be introduced, errors can be systematically planted, encounters can be standardized across learners, and outcomes can be measured without patient safety risk. The DEFT-AI supervisory structure - in which educators probe the trainee’s reasoning process, evaluate their use of supporting evidence, provide targeted feedback, and teach toward identified gaps - applies directly to post-simulation debriefing across all four domains. As a research venue, the same infrastructure enables the developmental studies clinical deployments cannot produce: comparative trials of trainees with versus without explicit AI documentation review curriculum, longitudinal tracking of documentation competency and clinical reasoning across training stages, and empirical determination of when in the training pipeline ambient AI should be introduced. Many academic medical centers already have simulation infrastructure that could be adapted, though capacity and access vary across institutions, specialties, and settings.

The timing of ambient AI introduction is critical to ensure trainees develop clinical reasoning before they begin editing AI output. This framework governs the introduction of ambient AI after foundational authoring is established, not authoring itself. Because independent authoring is how clinical reasoning is first built, the earliest stages of training should remain authoring-predominant, with ambient AI review introduced only once that reasoning is in place. Otherwise, trainees risk never-skilling rather than deskilling, becoming passive editors before they have been active authors at all. When this transition should occur, and what counts as established foundational authoring are empirical questions we propose for study; neither is currently standardized across curricula.

The competencies proposed here are structured around a paradigm that is stable even as the underlying tools change: clinical documentation generated by AI and reviewed by a human. As long as that paradigm holds, the core competencies remain relevant regardless of which model is in use. Errors must still be detected even as error rates decline; documentation must still be reconciled against the encounter, and independent reasoning must still be preserved. Trust calibration, however, carries an inherent temporal dimension. The specific conditions under which AI underperforms are not fixed; as models advance, the appropriate calibration can shift from teaching restraint against over-trust toward guarding against reflexive under-trust. The software-engineering shift from line-by-line review of AI completions toward verification of agentically produced outcomes illustrates the same trajectory. The durable competency is therefore not mastery of any one generation’s failure modes but the ability to recalibrate as tools change; trust calibration is best taught as an adaptive skill rather than a fixed checklist.

Taken together, three features distinguish this framework from existing supervision and guardrail models: its focus on ambient documentation review specifically rather than AI use in general; its organization of that competency into four teachable, assessable domains; and its use of simulation as a dual venue that both teaches the competency and generates the developmental evidence the clinical literature has not produced.

The competencies, their sequencing, and the assessment methods in Table 1 require empirical testing, as does the dual-function role we propose for simulation. Simulation settings, where encounters can be standardized and errors deliberately planted, make several questions answerable, including whether targeted training improves trainees’ error-detection rates on notes with planted errors, whether training with ambient AI erodes independent authoring ability measured under “AI-off” conditions, and when in training review can be introduced without displacing foundational authoring. This framework is also grounded in the U.S. medical education, regulatory, and documentation context, and application elsewhere will require adaptation. We leave formal study designs to future work; our aim is to define the competencies clearly enough that such studies become possible.

## Conclusion

The competencies required to use AI-generated clinical documentation safely invite reflection on what medical education has historically been asked to teach, and what it has not. Reviewing AI documentation may appear to be a small editorial task, yet it carries the cognitive functions - synthesis, prioritization, and judgment - that independent documentation has long developed in trainees. The framework we propose here is preliminary, and the simulation infrastructure positioned to teach these competencies is the same infrastructure that may produce the developmental evidence the clinical literature cannot. The value of prospective curricular integration lies less in the specifics of any single framework than in the willingness of medical education to recognize a new competency before, rather than after, it shapes the next generation of clinicians.
